# Biosorption of Copper in Swine Manure Using *Aspergillus* and Yeast: Characterization and Its Microbial Diversity Study

**DOI:** 10.3389/fmicb.2021.687533

**Published:** 2021-08-12

**Authors:** Yongkang Zhen, Mengzhi Wang, Yalan Gu, Xiang Yu, Khuram Shahzad, Jun Xu, Yuqing Gong, Peizhen Li, Juan J. Loor

**Affiliations:** ^1^College of Animal Science and Technology, Yangzhou University, Yangzhou, China; ^2^State Key Laboratory of Sheep Genetic Improvement and Healthy Production, Xinjiang Academy of Agricultural and Reclamation Sciences, Shihezi, China; ^3^Department of Biosciences, COMSATS University Islamabad, Islamabad, Pakistan; ^4^Institute for Quality and Safety and Standards of Agricultural Products Research, Jiangxi Academy of Agricultural Sciences, Nanchang, China; ^5^Jiangsu Provincial Station of Animal Husbandry, Nanjing, China; ^6^Mammalian Nutrition Physiology Genomics, Division of Nutritional Sciences, Department of Animal Sciences, University of Illinois at Urbana-Champaign, Urbana, IL, United States

**Keywords:** *Aspergillus*, swine manure, copper, microbial biosorption, 16S and ITS rRNA sequence

## Abstract

Dietary copper supplementation in the feed of piglets generally exceeds 250–800 mg/kg, where a higher quantity (>250 mg/kg) can promote growth and improve feed conversion. Despite the reported positive effects, 90% of copper is excreted and can accumulate and pollute the soil. Data indicate that fungi have a biosorptive capacity for copper. Thus, the objectives of the present experiment were to study the effects of adding different strains of fungi on the biosorptive capacity for copper in swine manure and to evaluate potential effects on microbiota profiles. *Aspergillus niger* (AN), *Aspergillus oryzae* (AO), and *Saccharomyces cerevisiae* (SC) were selected, and each added 0.4% into swine manure, which contain 250 mg/kg of copper. The incubations lasted for 29 days, and biosorption parameters were analyzed on the 8th (D8), 15th (D15), 22nd (D22), and 29th (D29) day. Results showed that after biosorption, temperature was 18.47–18.77°C; pH was 6.33–6.91; and content of aflatoxin B1, ochratoxin A, and deoxynivalenol were low. In addition, residual copper concentration with AN was the lowest on D15, D22, and D29. The copper biosorption rate was also highest with AN, averaging 84.85% on D29. Biosorption values for AO reached 81.12% and for SC were lower than 80%. Illumina sequencing of 16S and ITS rRNA gene revealed that fungal treatments reduced the diversity and richness of fungal abundance, but had no effect on bacterial abundance. *Unknown_Marinilabiliaceae*, *Proteiniphilum*, *Tissierella*, and *Curvibacter* were the dominant bacteria, while *Aspergillus* and *Trichoderma* were the dominant fungi. However, the added strain of *S. cerevisiae* was observed to be lower than the dominant fungi, which contained less than 0.05%. The Kyoto Encyclopedia of Genes and Genomes (KEGG) pathway enrichment predicted *via* PICRUSt2 that there were bacterial genes potentially related to various aspects of metabolism and environmental information processing. Overall, data indicated that *Aspergillus* can provide microbial materials for adsorption of copper.

## Introduction

It has been demonstrated through several studies that copper can accelerate the growth and development of livestock (Zhi et al., 2020), including broilers (Ewing et al., 1998), goats (Solaiman et al., 2001), and rabbits (Skrivanova et al., 2001). This trace mineral improves growth and feed conversion rates of piglets, thus enhancing production performance (Stahly et al., 1980; Apgar et al., 1995). At a mechanistic level, high copper stimulates the synthesis and secretion of growth-promoting hormone-related factors in piglets (Yang et al., 2011). Other studies revealed that high dietary copper increases activities of various digestive enzymes in the gastrointestinal tract of piglets, including pepsin and trypsin, accelerates gastrointestinal peristalsis, and improves feed digestibility (Luo and Dove, 1996). High dietary copper also increases the activity of antioxidant enzymes and accelerates gastric acid secretion in piglets, which is beneficial to health. Copper sources in animal feed include mainly copper sulfate, basic copper chloride, copper citrate, and chelated copper (Armstrong et al., 2000; Zhai et al., 2006). Thus, inclusion of high copper levels in feed has become a common practice, with compound feeds for growing pigs containing more than 250–1,000 mg/kg (Nicholson et al., 1999; Eneji et al., 2001). However, almost 90% of the ingested copper is released into the soil through manure because animals have a low absorption capacity for this mineral. This leads to the accumulation of copper in the soil.

Long-term use of manure and fertilizers with high copper levels increases the risk of soil pollution, which may further cause water and plant pollution and even harm human health (Jondreville et al., 2003; Bai et al., 2015). In order to reduce the risk of high copper excretion into feces, soil, and water, in 2018, the *Official Journal of the European Union* published a document (No. EU2018/1039) detailing the standards for the use of copper as a feed additive for all farmed animals. It decreased the copper limit for suckling pigs to 150 mg/kg and piglets to 100 mg/kg, which represented a reduction of 11.8 and 41.2% compared with previous values, respectively. The revision of the standard reflected a serious approach to ensuring environmental protection in the livestock industry. In fact, in order to continue developing efficient animal husbandry approaches to reduce pollution, there is a need for continued research to ensure sustainable development and environmental protection.

High copper removal technology for soil and sewage has been developed. The passivation of heavy metals (Wuana and Okieimen, 2011) using agents such as lime, phosphorous lime, biochar, sodium silicate, and attapulgite bar can reduce the bioavailability of soil copper (Zhang et al., 2011; Hale et al., 2012; Cui et al., 2016; Jones et al., 2016). Removal of copper from wastewater includes mainly physical, chemical precipitation, and biological methods. Biotechnological methods pertain to removal of copper *via* biological metabolism, adsorption, repair, and flocculation, mainly through cell surface adsorption and intracellular aggregation (Javanbakht et al., 2014; Osmanlioglu, 2018; Virolainen et al., 2019). Compared with the treatment in soil and sewage, treatment technologies for high copper in manure have been less studied.

Fungi such as *Aspergillus*, *Cladosporium*, and *Saccharomyces* can adsorb and remove copper and have been used previously (Chang et al., 1997; Sayer and Gadd, 1997; Gunasundari and Kumar, 2017). The process of biosorption is complex, with the first step being adsorption of copper to the active groups such as hydroxyl (–OH), carboxyl (–COOH), amino (–NH_2_), and sulfhydryl (–SH) on fungal cell walls and membranes (Dursun, 2006; Noormohamadi et al., 2019). In addition, polysaccharides on the cell wall secrete active groups and chitosan, further enhancing the adsorption capacity of copper (Zabochnicka-Swiatek and Krzywonos, 2014). Part of copper will enter the cell when the adsorption approaches saturation, and Cu^2+^ is reduced to Cu^+^ by *FRE1/2* gene and then transported into the cell through the copper transporter gene *CTR1/3* (Dancis et al., 1994; Georgatsou et al., 1997). Then, a copper chaperone protein will specifically bind to copper and transport it to various organelles, mainly through three pathways: (i) copper transporter cytochrome oxidase 17 (Cox17) receives Cu^+^ transported by CTR and sends it to the mitochondrial inner membrane (Heaton et al., 2000); (ii) the *Atx1* transfers copper to Ccc2p and binds to FET3p on the reverse side of the Golgi apparatus (Arnesano et al., 2001); and then (iii) copper chaperone CCS (copper chaperone for Sod1p) transports copper to Sod1p in cellular fluid to clean up superoxide ions (Schmidt et al., 1999). Furthermore, when copper is in excess, the glutathione system in fungal cells is activated to prevent copper stress (Zhu et al., 1999). Production of metallothionein and phytochelatins also aids in copper detoxification (Inouhe et al., 1996; Liti et al., 2009).

Previous reports indicated that *Saccharomyces cerevisiae* (Sun et al., 2016), *Aspergillus oryzae* (Long et al., 2017), and *Aspergillus niger* (Wang J.Y. et al., 2016) have a high efficiency for copper biosorption in water. Furthermore, they have a high degree of commercialization, are easy to reproduce, and are non-toxic. Because of these reasons, the above three strains were selected for testing the copper-absorbing capacity in swine manure. In addition, the sequencing data will reveal the interaction relationship between fungi and bacteria, and their superiority strains will be screened for post-production materials of copper adsorbent.

## Materials and Methods

### Selection of Fungi, Swine Manure, and Biosorption Conditions

*Saccharomyces cerevisiae* (SC), *A. oryzae* (AO), and *A. niger* (AN) were selected based on previous reports (Sun et al., 2016; Wang J.Y. et al., 2016; Long et al., 2017). Fungi were purchased from a local commercial company at >5 billion/g spores per fungi strain, cultured in media, and the spore concentration in the final suspensions determined by counting in a hemocytometer (Wang J.Y. et al., 2016). The experiment included a control (CON), SC, AO, and AN treatments, each replicated three times. Each treatment consisted of addition of 0.4% fungal spores to swine manure (4 g/kg) to ensure a density greater than 10^7^ cells/g, with a 10× density of liquid adsorption medium (Sun et al., 2019). Twenty-five kilograms fresh swine manure was collected from an experimental farm in which pigs were fed with full grain feed. Animals were free from clinical disease. The original copper concentration determined by an atomic absorption spectrophotometer (AAS, PinAAcle 900F, PerkinElmer Corporation, Waltham, MA, United States) in swine manure was 9.6 mg/kg (Nyantakyi et al., 2019). A total of 15.10 g copper sulfate was added to 10 L of deionized water, mixed, and transferred to 25 kg of manure to give a final copper content of 250 mg/kg. The manure was then divided into 12 subsamples, and 0.4% of each fungi strain mixed in. All vessels were then covered with a shielding net to avoid light and the biosorption simulated. The incubations lasted for 29 days, and biosorption parameters were analyzed on the 8th (D8), 15th (D15), 22nd (D22), and 29th (D29) day.

### Temperature, pH, and Mycotoxin Content

Manure samples on the final biosorption state (D29) were collected to determine the temperature, pH, and mycotoxin levels. During sampling, an alcohol thermometer was inserted into the manure at 5–10 cm to determine the temperature. Then, 9 mL distilled water was added into 1 g of manure and homogenized for 30 s to determine pH using a calibrated pHS-3C precision pH meter (Leimeg, Shanghai, China). Lastly, the concentration of mycotoxin was determined using the following methods according to manufacturer’s protocols: aflatoxin B1 test kit (CNW, SNEQ-C110119, Düsseldorf, Germany), ochratoxin A test kit (CNW, SNEQ-C11019, Düsseldorf, Germany), and deoxynivalenol test kit (CNW, SNEQ-C11002, Düsseldorf, Germany).

### Residual Copper Concentration and Copper Biosorption Rate

Air-dried swine manure samples were weighed to determine residual copper concentration and copper biosorption rate according to published protocols (Park et al., 2011; Wang W.Y. et al., 2016; Meier et al., 2017). Briefly, 5 g of air-dried samples was weighed, 20 mL deionized water was added, and the mixture was vortexed for 30 s. Then, samples were centrifuged at 8,000 rpm for 10 min, and the supernatant on the upper layer was then carefully absorbed and collected. This fraction contained the dissolved ionic copper from manure. Fungi in the middle layer were carefully removed and the precipitate at the bottom was collected. The supernatant was used to dissolve the manure precipitate again, repeating the above steps and washing the precipitate with deionized water. The mass of copper ions dissolved in swine manure was measured by AAS (PinAAcle 900F, PerkinElmer Corporation, Waltham, MA, United States). The sediment was dried at 60°C for 48 h for determining copper concentrations according to the published protocol (Nyantakyi et al., 2019). Residual copper concentration was the sum of the concentration of copper in supernatant and sediment. Copper biosorption rate was calculated by the following formula:

$$R\%=\frac{C_{0}-C_{i}}{C_{0}}\times 100\%$$

where *R*%, copper biosorption rate; *C*_0_, initial copper concentration; and *C*_*i*_, residual copper concentration (including the mass of copper in the supernatant and cleaning fluid).

### DNA Extraction, Illumina MiSeq Sequencing, and Data Processing

Total microbial DNA from manure samples in the CON, SC, AO, and AN groups was extracted using the fecal genome extraction kit (DP328, Tiangen Biotech Co., Ltd., Beijing, China) according to manufacturer’s protocols. The concentration and purity of total DNA (OD_260/280_ and OD_260/230_) were determined by the NanoDrop spectrophotometer (Thermo Fisher Scientific, Waltham, MA, United States). The integrity of DNA was determined by agarose gel electrophoresis.

Bacterial and fungal communities and diversities were determined by high-throughput sequencing with the Illumina Novaseq-PE250 platform at Genepioneer Biotechnologies Co., Ltd., Nanjing, China. Amplification of the V3–V4 region of bacterial rRNA genes was completed using the universal primers (319F: 5′-ACTCCTACGGGAGGCAGCAG-3′; 806R: 5′-GGACTACHVGGGTWTCTAAT-3′) (Yang et al., 2019). The ITS2 region of fungal rRNA genes was amplified with ITS3 and ITS4 primer (ITS3F: 5′-GCATCGATGAAGAACGCAGC-3′; ITS4R: 5′-TCCTCCGCTTATTGATATGC-3′) (Essel et al., 2019). The 16S and ITS rRNA gene amplicon sequencing data generated during the current study were submitted to NCBI under BioProject PRJNA704785.

Paired-end reads generated from Illumina platforms were processed and merged using FLASH software (v1.2.7) (Magoc and Salzberg, 2011). Then, high-quality clean sequence tags were obtained by removing lower quality and shorter lengths. Subsequently, the Uparse pipeline of Usearch (v7.1) software (Edgar, 2013) was employed to cluster all reads from each sample into operational taxonomic units (OTUs) at a 97% sequence similarity level. Representative sequences of each OTU were screened for further annotation. Taxonomic information was compared to the SILVA database (Release 115) (Quast et al., 2013) for bacterial 16S rRNA genes, while UNITE v8.0 database (Nilsson et al., 2019) was used for annotating fungal ITS rRNA gene reads. Taxonomies were grouped at the phylum, class, order, family, and genus levels. The bacterial and fungal community richness (Chao1 and ACE indexes), diversity (Shannon and Simpson indexes), and Good’s coverage were calculated with QIIME (v1.9.1) software (Caporaso et al., 2010). Linear discriminant analysis effect size (LEfSe) was used to elucidate bacterial and fungal genus classification taxa that were associated with each fungal treatment (Segata et al., 2011). LEfSe was calculated using the Galaxy web platform^1^, and only linear discriminant analysis (LDA) scores ≥2 were listed. The FAPROTAX software (Louca et al., 2016) was used to predict ecological functions of microbial communities. In addition, microbial community functions were predicted by PICRUSt2 (v1.1.0) based on high-quality sequences and annotated in the Kyoto Encyclopedia of Genes and Genomes (KEGG) database (Langille et al., 2013).

### Statistical Analysis

Temperature, pH, mycotoxins, residual copper concentration, copper biosorption rate, and alpha diversity index were subjected to the one-way ANOVA using SPSS 13.0 software. The line chart of residual copper concentration and copper biosorption rate was made by GraphPad Prism 6.0 software. Bacterial and fungal taxa analyses were plotted using the GraphPad Prism 6.0 software based on the modified OTU data. Only those taxa with an abundance >1% of the total community in at least one treatment were included. The Venn diagram was analyzed using the R package “VennDiagram.” Pearson’s correlation analysis was carried out by R package “ggplot2,” “corrplot,” and “pheatmap.” The bubble diagram of pathway enrichment analysis was made using the R package “ggplot2.” The R version was 4.0.2, and R packages were described previously (Chen and Boutros, 2011; Li et al., 2021). Significance was declared at *P* < 0.05.

## Results

### Temperature, pH, and Mycotoxin Concentrations

Temperature, pH, aflatoxin B1, ochratoxin, and deoxynivalenol of final biosorption state are reported in Table 1. The pH of CON and AN was significantly higher than that of SC (*P* < 0.05). The concentration of aflatoxin B1 and deoxynivalenol of CON was significantly higher than that of SC, AO, and AN (*P* < 0.05). No significant differences (*P* > 0.05) were observed in temperature and ochratoxin A among treatments. The temperature was close to the fermentation room temperature, and pH values were near the original pH of manure.

**TABLE 1:** The temperature, pH, aflatoxin B1, ochratoxin, and deoxynivalenol of final state.

Items	Treatments				SEM	*P*-value
	CON	SC	AO	AN		
Temperature, °C	18.48	18.47	18.57	18.77	0.13	0.883
pH	6.91^a^	6.33^b^	6.60^ab^	6.82^a^	0.09	0.037
Aflatoxin B1, μg/kg	3.33^a^	1.41^b^	1.08^b^	1.19^b^	0.31	0.002
Ochratoxin A, μg/kg	3.52	3.53	3.37	3.20	0.10	0.702
Deoxynivalenol, mg/kg	0.58^a^	0.25^b^	0.22^b^	0.18^b^	0.05	0.005

*CON, biosorption without adding fungi; SC, biosorption with adding S. cerevisiae; AO, biosorption with adding A. oryzae; AN, biosorption with adding A. niger; SEM, standard error of the mean. Means within a row with different superscripts are different at P < 0.05.*

### Residual Copper Concentration and Biosorption Rate

**FIGURE 1:**
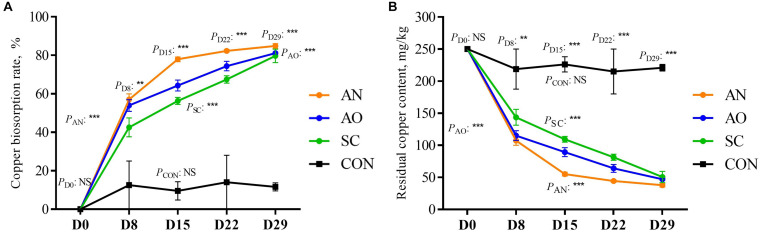
Residual copper concentration and biosorption rate of copper in swine manure on D8, D15, D22, and D29. **(A)** Curve diagram of residual copper content in swine manure in different treatments over different days. **(B)** Curve diagram of copper biosorption rate of swine manure in different treatments over different days. CON, biosorption without adding fungi; SC, biosorption with adding *S. cerevisiae*; AO, biosorption with adding *A. oryzae*; AN, biosorption with adding *A. niger*. **P < 0.01; ***P < 0.001; ^*NS*^*P* > 0.05.

The residual copper concentration and biosorption rate in swine manure on D8, D15, D22, and D29 are shown in Figure 1. Compared with different biosorption periods, except for CON, there were significant differences in copper biosorption rate and residual copper concentration in feces among the three treatments (*P* < 0.05). When studying the copper biosorption rate and residual copper concentration among different groups in the same day, treatments AN, AO, and SC were significantly higher than CON (*P* < 0.05). Overall, except with CON, residual copper concentration decreased continuously, while the biosorption rate increased from D0 to D29. Residual copper concentration for AN was the lowest on D15, D22, and D29, and copper biosorption rate was the highest, averaging 84.85%. Biosorption rate with AO reached 81.12% and with SC it averaged 80%.

### Bacterial and Fungal Sequencing OTUs and Alpha Diversities

**FIGURE 2:**
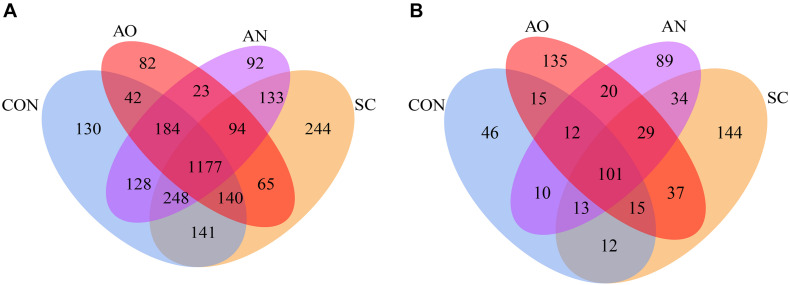
Venn diagram of manure bacterial and fungal operational taxonomic units (OTUs). The number of unique OTUs was represented by the unoverlapped portion of Venn diagram for each group. **(A)** Bacterial Venn diagram and **(B)** fungal Venn diagram.

The Venn diagram revealed that the numbers of unique OTUs for the CON, SC, AO, and AN were 130, 244, 82, and 92 of bacteria, respectively, and the shared OTU number was 1,177 (Figure 2A). For fungi, the unique OTU numbers were 46, 144, 135, and 89, respectively, and included 101 of shared OTUs (Figure 2B). The number of bacterial OTUs obtained by sequencing was much higher than that of fungi. In addition, detected OTU coverage index was greater than 99.70% for each treatment (Table 2).

**TABLE 2:** Numbers of observed species, richness, and alpha diversity indices in biosorption samples from each treatments groups.

Items	Treatments				SEM	*P*-value
	CON	SC	AO	AN		
**Bacteria**						
OTUs	1,859.00	2,076.67	2,103.67	1,821.33	60.49	0.230
Chao1 index	2,087.10	2,273.60	2,333.80	2,097.70	47.69	0.152
ACE index	2,075.80	2,285.60	2,337.50	2,123.60	45.79	0.108
Shannon index	6.41	7.37	7.39	6.81	0.16	0.064
Simpson index	0.95	0.98	0.98	0.96	0.00	0.137
Coverage (%)	99.74	99.76	99.73	99.71	0.02	0.871
**Fungi**						
OTUs	309.00^a^	345.33^a^	216.00^b^	222.00^b^	19.91	0.010
Chao1 index	333.79^a^	351.29^a^	220.36^b^	243.20^b^	18.73	0.002
ACE index	319.13^a^	350.23^a^	221.44^b^	239.08^b^	17.74	0.002
Shannon index	4.04^a^	4.71^a^	0.60^b^	1.64^b^	0.53	<0.001
Simpson index	0.75^a^	0.85^a^	0.15^c^	0.49^b^	0.09	0.001
Coverage (%)	99.85	99.99	99.97	99.97	0.03	0.185

*CON, biosorption without adding fungi; SC, biosorption with adding S. cerevisiae; AO, biosorption with adding A. oryzae; AN, biosorption with adding A. niger; SEM, standard error of the mean; OTUs, operational taxonomic units; ACE index, abundance-based coverage estimator. Means within a row with different superscripts are different at P < 0.05.*

In terms of bacterial alpha diversity (Table 2), compared with CON, fungal treatments did not change OTU numbers. No differences were observed in the Chao1, ACE, Shannon, and Simpson indexes among the CON and fungal treatments (*P* > 0.05). However, for fungal alpha diversity, AO and AN treatments decreased OTU numbers, Chao1, ACE, Shannon, and Simpson indexes compared with CON and SC treatments (*P* < 0.05).

### Bacterial and Fungal Community Abundance at Different Levels

**FIGURE 3:**
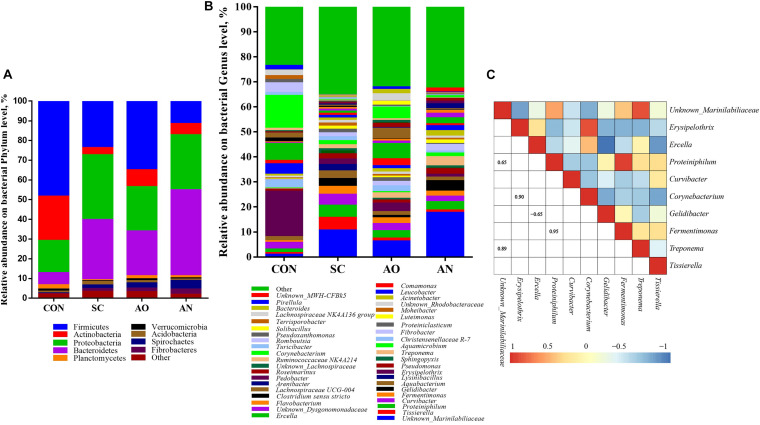
Taxonomic composition of bacterial communities associated with different fungal treatments. **(A)** The relative abundance of bacteria at phylum level; **(B)** the relative abundance of bacteria at genus level; **(C)** Pearson correlations of samples with the OTU abundance at genus in the relative abundance of top 10. Blue boxes represent negative correlations, and red boxes represent positive correlations between microbes. OTUs that were < 1% of the average relative abundance in groups are summarized as “other”.

Figure 3 underscores that the species of bacteria were different at the phylum and genus levels. The taxonomic analysis at the phylum level revealed that bacterial communities were predominantly Firmicutes, Bacteroidetes, Actinobacteria, and Proteobacteria (Figure 3A). At the genus level, the dominant groups were *Unknown_Marinilabiliaceae*, *Proteiniphilum*, *Gelidibacter*, and *Fermentimonas* of phylum Bacteroidetes; *Erysipelothrix*, *Ercella*, *Tissierella*, and *Christensenellaceae R-7* of phylum Firmicutes; *Corynebacterium* and *Leucobacter* of phylum Actinobacteria; *Curvibacter* of the phylum Proteobacteria; and *Treponema* of the phylum Spirochaetes (Figure 3B). A heatmap correlation analysis was established based on significant correlations among different bacteria for the top 10 genera (Figure 3C). The high abundance of *Unknown_Marinilabiliaceae* exhibited co-occurrence correlations with *Proteiniphilum* (*R* = 0.65) and *Treponema* (*R* = 0.89), *Erysipelothrix* exhibited co-occurrence correlations with *Corynebacterium* (*R* = 0.90), and *Proteiniphilum* exhibited co-occurrence correlations with *Fermentimonas* (*R* = 0.95), whereas it showed a negative interaction between *Gelidibacter* and *Ercella* (*R* = −0.65).

**FIGURE 4:**
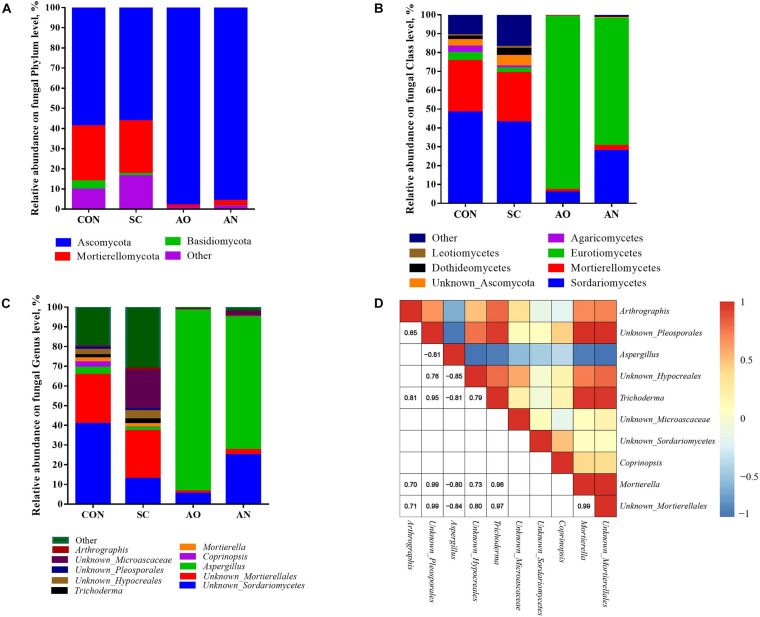
Taxonomic composition of fungal communities associated with different fungal treatments. **(A)** The relative abundance of fungi at phylum level; **(B)** the relative abundance of bacteria at class level; **(C)** the relative abundance of fungi at genus level; **(D)** Pearson correlations of top 10 samples with the OTU abundance at genus level. Blue boxes represent negative correlations, and red boxes represent positive correlations between microbes. OTUs that were <1% of the average relative abundance in groups are summarized as “other”.

The relative abundance of fungal communities among the four groups was examined at phylum, class, and genus levels, and the taxonomy of different levels varied among the four groups (Figure 4). Ascomycota, Mortierellomycota, and Basidiomycota mainly dominated the fungal communities at the phylum level (Figure 4A). The relative abundances of Ascomycota in AO and AN were 98.08 and 95.87%, respectively. At the class level, the dominant groups mainly included Sordariomycetes, Eurotiomycetes, Unknown_Ascomycota, Dothideomycetes, and Leotiomycetes of the phylum Ascomycota; Mortierellomycetes of the phylum Mortierellomycota; and Agaricomycetes of the phylum Basidiomycota (Figure 4B). Furthermore, at the genus level, the class Eurotiomycetes was represented by *Aspergillus*; the class Sordariomycetes was represented by *Unknown_Sordariomycetes*, *Trichoderma*, *Unknown_Microascaceae*, and *Unknown_Hypocreales*; and the class Mortierellomycetes was represented by *Mortierella* and *Unknown_Mortierellales* (Figure 4C). The heatmap correlation analysis of the top 10 fungal genera showed that the high abundance of *Aspergillus* had negative interactions with other fungal genera, while other genera mainly presented co-occurrence correlations among each other, which explained why *Aspergillus* present abundantly. Saccharomycetes only accounted for less than 0.05% of each group.

### Taxonomic Differences in Microbial Genus Level With AO and AN

**FIGURE 5:**
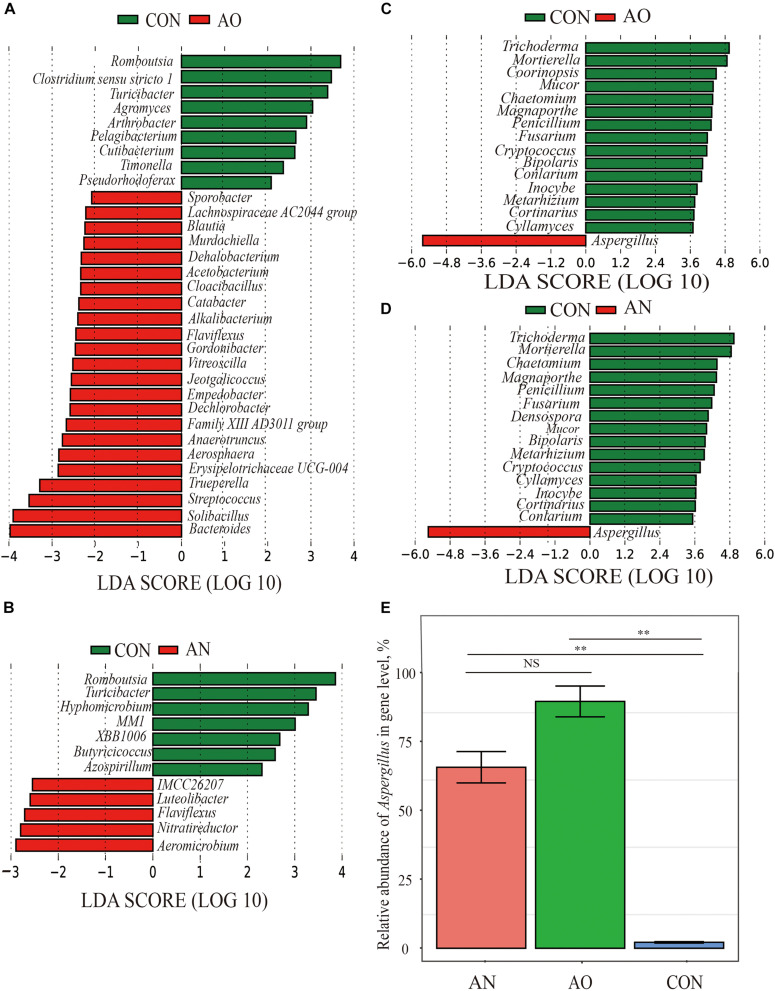
Screening of significant taxonomic differences in microbial genus level of AO and AN using LEfSe. **(A)** Linear discriminant analysis (LDA) plus effect size (LEfSe) at bacterial genus levels of CON and AO; **(B)** LEfSe at bacterial genus levels of CON and AN; **(C)** LEfSe at fungal genus levels of CON and AO; **(D)** LEfSe at fungal genus levels of CON and AN. The LDA score that was higher in the CON is shown in green, whereas one LDA score that was elevated fungal treatments is depicted in red. Only LDA scores > 2 were listed. **(E)** Comparative histogram of relative abundance of *Aspergillus* in AN, AO, and CON. **P < 0.01, ^*NS*^*P* > 0.05.

Significant taxonomic differences in bacterial and fungal genus classification between CON, AO, and AN groups were analyzed using LEfSe (Figure 5). LEfSe results were visualized using taxonomy bar chart. At the bacterial genus level between CON and AO (Figure 5A), *Romboutsia*, *Clostridium sensu stricto 1*, *Turicibacter*, and *Agromyces* were enriched in CON, whereas *Bacteroides*, *Solibacillus*, *Streptococcus*, and *Trueperella* were enriched in AN. Between CON and AN (Figure 5B), *Romboutsia*, *Turicibacter*, *Hyphomicrobium*, etc., were enriched in CON, whereas *Aeromicrobium*, *Nitratireductor*, and *Flaviflexus* were enriched in AN. At the fungal genus level between CON and AO (Figure 5C), *Trichoderma*, *Mortierella*, and *Coprinopsis* were enriched in CON, whereas *Aspergillus* was enriched in AO. Between CON and AN (Figure 5D), *Trichoderma, Mortierella*, and *Chaetomium* were enriched in CON, whereas *Aspergillus* was enriched in AN. The relative abundances of *Aspergillus* in AO and AN was 91.69 and 67.24%, respectively (Figure 5E), which were significantly higher than those of CON (2.20%) (*P* < 0.01), but no significant difference was found between AO and AN (*P* > 0.05).

### Co-occurrence of Bacteria and Fungi in the Copper Biosorption System

**FIGURE 6:**
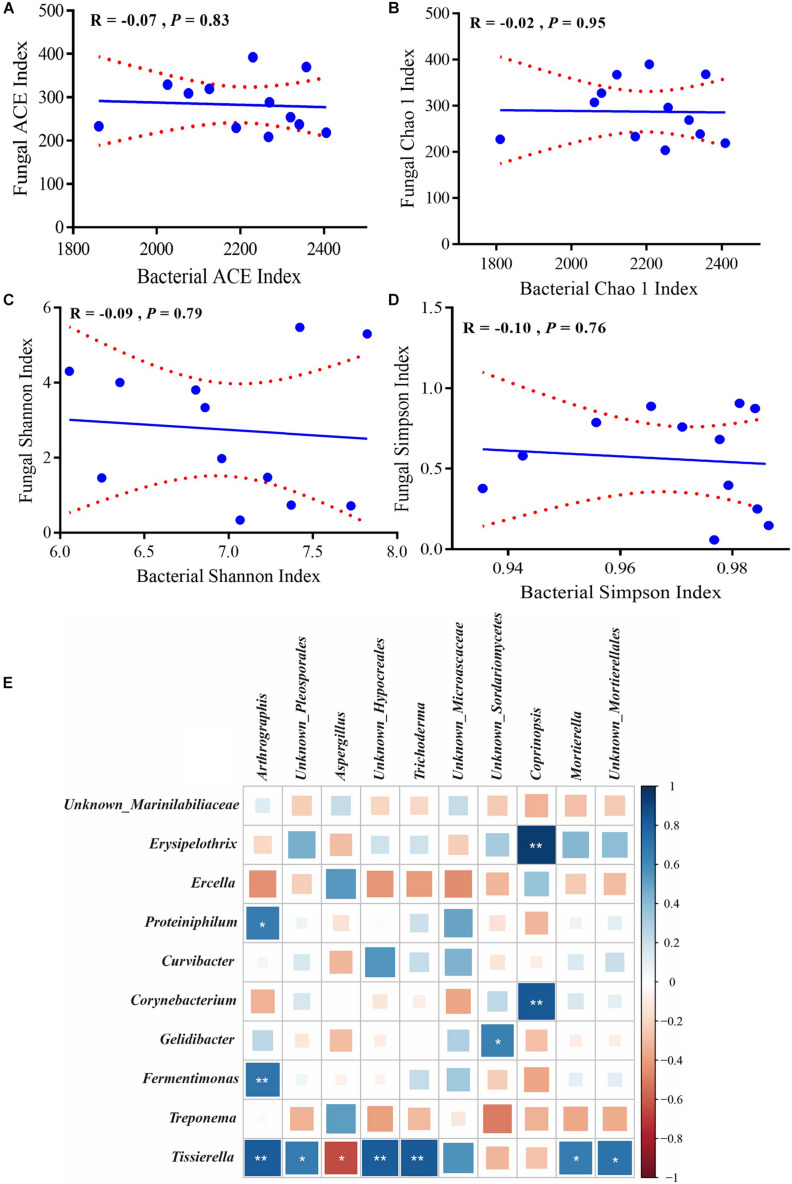
Correlation of bacterial and fungal alpha diversity. **(A)** ACE, **(B)** Chao1, **(C)** Shannon, and **(D)** Simpson indexes. The Pearson correlation coefficient (*R*) and the significance level (*P*) were shown on the plots. **(E)** Pearson correlations of samples with the OTU abundance of top 10 bacterial and fungal genera. Positive correlations are displayed in blue and negative correlations are shown in red. Color intensity and the size of the squares are proportional to the correlation coefficients. * Means significant correlations with | *R* | > 0.5 and *P* < 0.05 while ** Means significant correlations with | *R* | > 0.5 and *P* < 0.01.

The alpha diversity indexes and top 10 genera were used to determine correlations between bacteria and fungi. Figures 6A–D underscore that both species richness (Chao1 and ACE indexes) and species diversity (Shannon and Simpson indexes) were not significantly correlated (*P* > 0.05). In addition, the relative abundance of *Tissierella* was positively correlated with *Arthrographis*, *Unknown_Pleosporales*, *Unknown_Hypocreales*, and *Trichoderma* (*R* > 0.5, *P* < 0.05) but was negatively correlated with *Aspergillus* (*R* < −0.5, *P* < 0.05); the relative abundance of *Fermentimonas* and *Proteiniphilum* was positively correlated with *Arthrographis* (*R* > 0.5, *P* < 0.05); the relative abundance of *Corynebacterium* and *Erysipelothrix* was positively correlated with *Coprinopsis* (*R* > 0.5, *P* < 0.05) (Figure 6E).

### Prediction of Bacterial Functions via FAPROTAX or PICRUSt2

**FIGURE 7:**
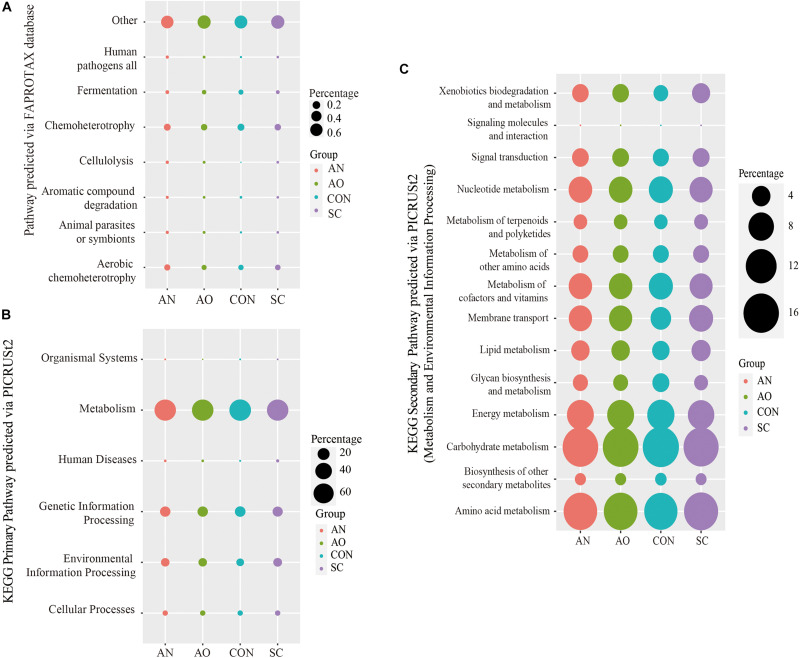
Prediction of bacterial functions *via* FAPROTAX or PICRUSt2. **(A)** The bacterial community ecological functions investigated *via* FAPROTAX; **(B)** the bacterial functional predictions of the Kyoto Encyclopedia of Genes and Genomes (KEGG) primary pathways; **(C)** the bacterial functional predictions of KEGG secondary pathways *via* metabolism and environmental information processing.

The bacterial community ecological functions were investigated via FAPROTAX (Figure 7A). The results indicated that the bacterial community contained a high number of sequences assigned to chemoheterotrophy, aerobic chemoheterotrophy, and fermentation. Other ecological functions such as cellulolysis, animal parasites or symbionts, human pathogens, and all aromatic compound degradation were also predicted to be present among bacterial communities in relatively lower abundance. However, nearly 60% of the sequencing annotations could not be classified by FAPROTAX.

The bacterial functional predictions of KEGG pathways based on PICRUSt2 are shown in Figures 7B,C. The results showed that the genes identified are potentially related to metabolism, genetic information processing, and environmental information processing with other pathways, including cellular processes, organismal systems, and human diseases presented in relatively lower abundance (Figure 7B). Based on metabolic pathways, most of the predicted genes are involved in carbohydrate metabolism, amino acid metabolism, energy metabolism, nucleotide metabolism, and metabolism of cofactors and vitamins (Figure 7C). In addition, another pathway related to this study with a high abundance and environmental information processing had genes predicted to encode membrane transporters and signal transduction proteins (Figure 7C).

## Discussion

Detection of mycotoxins such as aflatoxin, ochratoxin, and deoxynivalenol is important to evaluate the biosafety of manure after biosorption of copper (Table 1). Aflatoxin is a metabolite produced by *Aspergillus flavus*, *Aspergillus parasiticus*, and other fungi, mainly including AFB1, AFB2, AFG1, and AFG2, among which AFB1 is the most toxic and harmful (Das et al., 2014). After crops are contaminated by AFB1 and consumed by animals, the toxins will be transmitted along with the food chain and eventually threaten the safety of human beings. Similarly, in the process of adding fungi to adsorb copper, if some fungi in manure produce AFB1, it will inevitably cause mycotoxin accumulation pollution after the production of organic fertilizer. The AFB1 of CON was significantly higher than that of SC, AO, and AN, and this is because the *Aspergillus* added in manure detoxified the AFB1, for example, bacteria such as *Bacillus licheniformis* (Rao et al., 2017) and *Pseudomonas aeruginosa* (Samuel et al., 2014) and fungi such as *A. niger* (Xu et al., 2013) and *Zygosaccharomyces rouxii* (Zhou et al., 2017) can degrade 80–90% of AFB1. In addition, ochratoxin is another kind of mycotoxin found after aflatoxin. It is the metabolite of *Aspergillus* and *Penicillium*, such as *Aspergillus ochre*, *A. niger*, and *Penicillium viridicatum* (Puntaric et al., 2001). Among three types of ochratoxin (OTA, OTB, and OTC), OTA is the most toxic (Galtier et al., 1981). The concentrations of OTA were lower than 4.0 μg/kg among fungal treatments and CON, suggesting that it is not produced in the biosorption process. Deoxynivalenol is another mycotoxin mainly produced by the metabolism of Fusarium that, at low doses, can lead to anorexia, vomiting, diarrhea, and fever in animals, and it is also harmful to the immune system (Pinton et al., 2010). The deoxynivalenol concentration of fungal treatments was below 0.25 mg/kg, which was significantly lower than CON. It is possible that *S. cerevisiae* (Repeckiene et al., 2013), *A. oryzae* (Garda-Buffon et al., 2011), or some anaerobic bacteria and bacillus degraded deoxynivalenol. Overall, the concentration of mycotoxins in swine manure going through fermentation was extremely low, suggesting it is safe for production of bio-organic fertilizer or bedding.

The low production cost, high adsorption efficiency, and wide range of usage render biosorption of heavy metals using bacteria and fungi one of the most common methods to reduce pollution in manure (Solisio et al., 2006; Lodi et al., 2008). The most effective approach in the present experiment was *A. niger*, 0.4% addition with a copper concentration of 250 mg/kg and a biosorption rate of 84.85%. *A. oryzae* also had an adsorption rate of over 80%, which was due to the characteristics of *Aspergillus* themselves (Figure 1). Price et al. (2001) found that *A. niger* is capable of removing 91% of copper and 70% of zinc from swine wastewater. Proteomic analysis of *A. niger* showed that its capacity to absorb metals was boosted by physiological modification under stress, more specifically antioxidant responses induced by activity of catalase, superoxide dismutase, and cytochrome c peroxidase (Dias et al., 2019). Luna et al. (2015) found that exposing *A. niger* cells to increasingly large concentrations of copper led to ultrastructural changes in the cell surface, electron density, thickness, and septation. In addition, this strain has strong adaptability to the environment, a wide range of culture substrate sources, and the spores, and aerial hyphae produced also have biosorption capacity (Wang and Cui, 2017). Amounts of fungi as low as 0.4% were effective to elicit a strong biosorption effect; thus, only small amounts of these microorganisms would be needed in practice. One reason for this strong effect is that the biosorption capacity of metals increases rapidly at the beginning, and after reaching the equilibrium point, the biosorption capacity decreases with addition of the strain (Sag and Kutsal, 2000). Thus, if more strain was added above the 0.4% used in the present study, it might have led to a decrease in biosorption rate. When using *A. niger* to adsorb copper, Mukhopadhyay et al. (2007) found that the amount of metal ions adsorbed by fungi per gram decreased with the increase of strain biomass. Increased biomass interfered with the binding sites between metal ions and the strain. The adsorbed metal ions block the voids on the cell surface or agglomerate between ions, leading to reduced availability of active sites. The enrichment of high biomass will form a shielding effect in the outer layer, thus reducing the biosorption of metal ions.

The temperature was between 18.47 and 18.77°C, while pH was between 6.33 and 6.91 in the biosorption system and was close to the neutral pH and indoor temperature (Table 1). It has been reported that pH also affects the biosorption efficiency. A slightly acidic pH is more suitable for fungi to absorb heavy metals; under this acidic environment, fungi have the highest biological activity, the best quality of mycelium and spores, and the strongest negative charge on the surface of fungal cells, which are more conducive to the positive ions (Sepehr et al., 2012). Kapoor and Viraraghavan (1997) adsorbed lead ion, nickel ion, and cobalt ion with *A. niger*, and the results showed that, to a certain extent, the higher the pH value, the higher the biosorption rate, and no biosorption was observed when the pH was 3.0. The biosorption capacity was at maximum when the pH increased between 4 and 6, as the pH affected the metabolic activity of the fungi. The pH between 5 and 5.5 is favorable for sorption of pollutants especially heavy metals by *Aspergillus* sp., whereas *S. cerevisiae* shows tolerance and adaptation at pH 2.5–4.5. One of the possible reasons for the low relative abundance of *S. cerevisiae* in this experiment may be that the pH was not suitable for its growth. Thus, optimal conditions of pH should be considered when using fungi to develop high copper adsorbents. In addition, in the manure composting industry, *A. niger* can also turn macromolecules into small molecules and decompose insoluble substances into easy-to-volume substances. The physical and chemical properties of fertilizer have been greatly improved in the process of composting, thus increasing the economic benefit of the fertilizer and leading to higher forage yield (Du et al., 2020). Clearly, using *A. niger* can not only effectively remove copper in feces but also improve the commercial value of manure as organic fertilizer, underscoring the potential for practical application of this strain.

For alpha diversity of bacteria, there were no significant differences between Chao1, ACE, Shannon, and Simpson indexes (*P* > 0.05), suggesting that the species richness and diversity in the bacterial community were consistent among the four groups. Fungal treatment had no effect on bacterial community (Table 2). In contrast, compared with the CON and *S. cerevisiae* treatments, *A. oryzae* and *A. niger* treatments decreased the OTU numbers and Chao1, ACE, Shannon, and Simpson indexes (*P* < 0.05) (Table 2). This suggested that fungal treatments reduced the diversity and richness of fungal population, but had no effect on bacterial population. *A. oryzae* and *A. niger* addition significantly increased the proportion of *Aspergillus*. The proportion of *S. cerevisiae* was observed abnormally lower to be the dominant fungi for biosorption (accounted for <0.05%). Compared with CON, the addition of *S. cerevisiae* did not change the fungal community, but after addition of *A. niger* and *A. oryzae*, these two fungi grew and propagated rapidly, which inhibited the activity of other bacteria and fungi such as *Tissierella*, *Unknown_Hypocreales*, *Trichoderma*, and *Mortierella* (Figures 4D, 6E). This effect caused a reduction of the richness and diversity of species. The specific reasons for the failure of *S. cerevisiae* to grow need further analysis. It is possible that some antibiotics, traditional Chinese medicine preparations, and antifungal agents in swine manure inhibited its growth including phenolic and phenolic acids (Adeboye et al., 2014). In addition, some other fungi competed to inhibit *S. cerevisiae* growth or the biosorption environment was not suitable to support active growth of *S. cerevisiae.*

*Bacteroides*, *Solibacillus*, *Streptococcus*, *Aeromicrobium*, *Nitratireductor*, and *Nitratireductor* were the dominant bacteria in the *Aspergillus* treatments; fungal dominant species only contained *Aspergillus* (Figure 5). These bacteria genera mainly belong to Bacteroidetes, Firmicutes, and Proteobacteria phyla. They are the dominant phyla taxonomy in the gastrointestinal tract and manure of swine (Yang et al., 2016). Bacteroidetes and Firmicutes phyla are mainly related to carbohydrate metabolism, while Proteobacteria phyla are mostly related to protein metabolism (Lamendella et al., 2011; Regmi et al., 2011). This is consistent with our findings on the prediction of bacterial pathways (Figure 7). Results of bacterial community ecological function analysis *via* FAPROTAX indicated that bacteria were mainly associated with chemoheterotrophy, aerobic chemoheterotrophy, and fermentation. In addition, the bacterial functional predictions of KEGG pathways based on PICRUSt2 showed that genes were potentially related to carbohydrate metabolism, amino acid metabolism, energy metabolism, and nucleotide metabolism. The leading role of bacteria in the adsorption process is to decompose fecal organic matter, where the metabolism produces various kinds of nutrients. The dominant bacteria have strong degradation ability and mainly play a role in accelerating manure decay, decomposing toxic substances, and shortening the time of making organic fertilizers. It is noteworthy that some bacteria also have an ability to adsorb metal ions. The extracellular polysaccharides produced by *Lactococcus lactis* of Firmicutes phylum, *Methylobacterium organophilum* of Proteobacteria phylum, and *Anabaena spiroides* of Cyanobacteria phylum are an essential component of copper tolerance and biosorption (Gupta and Diwan, 2017; Kou et al., 2018). In addition, *Nostoc punctiforme* and *Cyanobacterium Synechococcus* of Cyanobacteria and *Pseudomonas putida* of Proteobacteria phylum can also produce metallothioneins to protect against metal toxicity (Hudek et al., 2009; Gupta and Diwan, 2017). Kou et al. (2018) concluded that *Methylobacillus*, *Solirubrobacter*, *Ohtaekwangia*, *Polaromonas vacuolata*, *Yonghaparkia alkaliphila*, and *Nitrosospira* are adapted to high metal concentrations and thus can be used readily in metal-treated soils. The biosorption rate of *S. cerevisiae* reached 77.92%, which might be due to the reason that *Trichoderma* of Sordariomycetes absorbed a portion of the added copper. *Trichoderma* also have strong biosorption of copper and have been widely used in practice. Furthermore, *Trichoderma* can also produce antibacterial proteins, which can not only kill pathogens in soil or feces through certain biosorption carriers but also improve nutrient utilization efficiency and repair agrochemical-polluted environments (Schuster and Schmoll, 2010). The above dominant microorganisms work together, where bacteria promote manure fermentation and fungi absorb copper and reduce copper pollution, both of which are of high practical value in production settings.

## Conclusion

In conclusion, *A. niger* and *A. oryzae* at a concentration of 0.4% and 250 mg/kg copper resulted in the greatest average copper biosorption rate, ∼85%. Both strains had a remarkable effect on reducing copper, and further optimization of parameters along with the use of embedding materials can lead to the development of an adsorbent for practical application. Although fungal treatments reduced the diversity and richness of fungal abundance, they had no effect on bacterial abundance. Overall, there may be multiple mechanisms acting synergistically to biotransform and adsorb copper so as to maintain a relatively stable state of copper ions in fungal cells. More studies are needed to better understand the specific pathways of copper biosorption by fungi such as *Aspergillus*.

## Data Availability Statement

The datasets presented in this study can be found in online repositories. The names of the repository/repositories and accession number(s) can be found below: https://www.ncbi.nlm.nih.gov/, BioProject PRJNA704785.

## Author Contributions

MW, YZ, YuG, KS, and JL designed the experiments and revised the manuscript. YZ, YaG, and XY conducted the experiments. MW, YuG, PL, and JX offered the experimental reagents and materials. YZ analyzed the data and finished the manuscript. YZ, MW, KS, and JL prepared the figures and edited the manuscript. All authors reviewed the manuscript.

## Conflict of Interest

The authors declare that the research was conducted in the absence of any commercial or financial relationships that could be construed as a potential conflict of interest.

## Publisher’s Note

All claims expressed in this article are solely those of the authors and do not necessarily represent those of their affiliated organizations, or those of the publisher, the editors and the reviewers. Any product that may be evaluated in this article, or claim that may be made by its manufacturer, is not guaranteed or endorsed by the publisher.

## Footnotes

1
https://huttenhower.sph.harvard.edu/galaxy/
